# Prognostic Value of the Pre-treatment Albumin-to-Alkaline Phosphatase Ratio in Patients With Metastatic Colorectal Cancer

**DOI:** 10.7759/cureus.102758

**Published:** 2026-01-31

**Authors:** Hacı Arak, Ercan Gumusburun, Havva Yesil Cinkir

**Affiliations:** 1 Medical Oncology, Gaziantep City Hospital, Gaziantep, TUR; 2 Medical Oncology, Faculty of Medicine, Gaziantep University, Gaziantep, TUR

**Keywords:** albumin-to-alkaline phosphatase ratio, colorectal cancer, neutrophil-to-lymphocyte ratio, platelet-to-lymphocyte ratio, prognostic value

## Abstract

Objective: This study aimed to evaluate the prognostic significance of the albumin-to-alkaline phosphatase ratio (AAPR) in patients with metastatic colorectal cancer (mCRC) and compare it with the neutrophil-to-lymphocyte ratio (NLR) and platelet-to-lymphocyte ratio (PLR) in the same patient cohort.

Methods: This retrospective study included patients who were followed for mCRC and whose pre-treatment albumin, alkaline phosphatase (ALP), NLR, and PLR values ​​were obtained. Receiver operating characteristic (ROC) curve analysis was performed to determine the optimal AAPR, NLR, and PLR cut-off values.

Results: This study included 182 patients. The patients’ median age was 57 (18-87) years, 110 (60.4%) were men, 162 (89%) had de novo metastatic disease, and 75 (41.2%) had right colon cancer. ROC analysis for the pre-treatment AAPR value of OS yielded an AUC of 0.622 (95% confidence interval (CI): 0.548-0.693, p=0.040), and the optimal AAPR cut-off value was ≤0.323. The median AAPR was lower in patients with liver metastasis than in those without (p=0.018) and in de novo metastatic patients than in recurrent patients (p=0.001). Median progression-free survival was longer in recurrent patients than in de novo metastatic patients (11 vs. 7 months, p=0.018). Univariate analysis showed that the AAPR significantly predicted OS (hazard ratio: 0.23,95% CI: 0.08-0.62, p=0.004), whereas NLR and PLR did not (p=0.718 and p=0.403, respectively). Median OS was 16 months (95% CI: 12.7-19.3) in all patients, 11 (95% CI: 7.5-14.5) months in the low-AAPR group, and 21 (95% CI: 17.4-24.6) months in the high-AAPR group (p=0.008).

Conclusion: In patients with mCRC, the OS was worse in the low-AAPR group than in the high-AAPR group. The AAPR showed a stronger univariable association with OS than NLR/PLR in this cohort, but did not retain independent significance in multivariable analysis; prospective validation is needed.

## Introduction

Colorectal cancer (CRC) ranks as the third most prevalent cancer and the second leading cause of cancer-related mortality globally [1]. Approximately 20% of colorectal cases are diagnosed at the metastatic stage. Approximately 50% of patients diagnosed at the local stage progress to the metastatic stage over time. The five-year survival rate of patients with metastatic colorectal cancer (mCRC) is approximately 5-10%. The most common metastatic sites include the liver, lungs, distant lymph nodes, peritoneum, bone, and brain [2].

Patients with mCRC comprise a heterogeneous group with different survival rates. Factors, such as patient age, performance status, comorbidities, tumor burden, metastatic sites, tumor localization, RAS, BRAF, microsatellite instability (MSI) status, and human epidermal growth factor receptor-2 (HER2) status, affect treatment decisions and survival [3]. The treatment predictive value of RAS status, HER2 status, BRAF status, MSI status, and the prognostic importance of BRAF status and tumor localization are known. However, these molecules provide information regarding the treatment response or survival of patients whose changes are detected [4]. The consensus molecular subtype (CMS) classification, derived from the genomic and epigenetic features of CRC, predicts the risk of recurrence and contribution of adjuvant therapy in early-stage patients and offers insights into treatment responses and prognosis in metastatic disease. However, in clinical practice, CMS classification is costly and not yet recommended in treatment guidelines [5]. Therefore, there is a need for inexpensive, accessible, and easily implementable prognostic tools applicable to all patients with mCRC.

Albumin (ALB), synthesized by hepatocytes, indirectly indicates liver function, nutritional status, and the immune system. Alkaline phosphatase (ALP), a group of isoenzymes frequently found in the liver, bones, and kidneys, catalyzes the removal of phosphate groups from nucleic acids. The prognostic importance of the albumin-to-alkaline phosphatase ratio (AAPR) has been demonstrated for some cancers [6]. Zhou et al. reported that overall survival (OS) increased as the pre-treatment AAPR rate increased in 808 patients with metastatic non-small cell lung cancer [7]. Li et al. found that OS and progression-free survival (PFS) were significantly different between metastatic gastric cancer patients with low and high AAPRs [8]. Cai et al. reported that the AAPR predicted OS in patients with advanced-stage hepatocellular cancer [9]. However, the prognostic importance of the AAPR in mCRC remains unexplored. Inflammation-related biomarkers, such as the neutrophil-to-lymphocyte ratio (NLR) and platelet-to-lymphocyte ratio (PLR), which can be obtained from routine blood examinations, have demonstrated predictive and prognostic use in mCRC and other malignancies [10].

This study aimed to comprehensively investigate the prognostic significance of the AAPR index in patients with mCRC and compare the prognostic significance of the AAPR, NLR, and PLR indices in the same patient group.

## Materials and methods

Study design

This retrospective, single-center study was conducted in accordance with the Declaration of Helsinki. This study retrospectively reviewed patients’ previous routine blood tests. No new blood or tissue samples were obtained from the patients, nor were the collected blood and tissue samples re-examined. Only the blood results were retrieved from the records and analyzed retrospectively. Furthermore, approximately 80% of these patients had died; therefore, the ethics committee waived the need to obtain informed consent. This study received no external funding and was approved by the Ethics Committee of Gaziantep University Faculty of Medicine (No.2023/279).

The inclusion criteria were as follows: patients with colorectal adenocarcinoma aged > 18 years, de novo metastasis at diagnosis or recurrent metastasis during follow-up, those who had not previously received systemic treatment for the diagnosis of metastatic cancer, who had serum ALB, ALP, and hemogram examinations performed within five days before systemic treatment, and those with follow-up data in our hospital system. The exclusion criteria were as follows: patients with known liver cirrhosis, kidney or bone disease, second primary cancer, active infection, or decompensated heart failure, and those whose data could not be accessed at our hospital. Patients who underwent surgery for ileus or perforation at diagnosis and those who died owing to surgical complications without receiving chemotherapy were excluded. This study included 182 patients who met the inclusion criteria and were diagnosed between June 2011 and May 2022.

Variables and definitions

Sex, age at diagnosis, performance score, date of diagnosis, tumor localization, lymphovascular invasion (LVI), perineural invasion (PNI), tumor grade, RAS and BRAF mutation status, patient metastatic sites, serum ALB level, ALP level, tumor markers, hemogram, first chemotherapy start date, and progression date were scanned in the patients' files or from the hospital electronic system. The presence of liver, lung, non-regional lymph node, or peritoneal metastasis was determined based on contrast-enhanced cross-sectional imaging or PET-CT results. The AAPR was calculated using the serum ALB to serum ALP level ratio. AAPR = albumin / alkaline phosphatase. The units for ALB and ALP are g/L and U/L, respectively. The NLR was defined as the number of neutrophils divided by the number of lymphocytes in the pre-treatment hemogram. The PLR was defined as the number of platelets divided by the number of lymphocytes.

PFS was defined as the time from the start date of the first chemotherapy to the date of progression, last follow-up, or death, whichever occurred first. OS was defined as the time from the date of diagnosis to the date of the last follow-up or death.

Statistical analysis

The normal distribution of the numerical data was tested using the Shapiro-Wilk test. Student's t-test was used to compare variables with normal distribution in the two groups, and the Mann-Whitney U test was used to compare variables that were not normally distributed in the two groups. Relationships between categorical variables were tested using the chi-square test. The relationships between the numerical variables were tested using correlation coefficients. Categorical data were presented as percentages. Continuous variables were expressed as median (minimum-maximum) or median (interquartile range (IQR)). Parameters with missing data, such as BRAF, KRAS, NRAS, and lung metastasis, were included only in descriptive statistics. Missing data were excluded from the analysis. Receiver operating characteristic (ROC) curve analysis and the Youden index were used to determine the optimal AAPR, NLR, and PLR cut-off values. Patients below the cut-off value were included in the "low group,” and those above the cut-off value were included in the "high group.” The Kaplan-Meier method and log-rank test were used to analyze the PFS and OS of the different groups. Uni- and multivariate analyses were conducted on variables affecting OS or PFS using the Cox proportional hazards regression model. IBM SPSS Statistics for Windows, Version 22 (Released 2013; IBM Corp., Armonk, New York, United States) was used for all analyses. P<0.05 was considered statistically significant.

## Results

Patient characteristics

This study included 182 eligible patients with mCRC. The patients’ median age was 57 (IQR, 47-68) years. Of these patients, 110 (60.4 %) were men, 162 (89%) had de novo metastasis, and 75 (41.2%) had right colon cancer. The patients’ baseline characteristics, including age at diagnosis, sex, Eastern Cooperative Oncology Group (ECOG) performance score, tumor localization, LVI, PNI, degree of tumor differentiation, liver metastasis, lung metastasis, peritoneal metastasis, and number of metastases, are shown in Table 1.

**Table 1:** Baseline characteristics of patients and distribution of baseline characteristics in patients with low-AAPR and high-AAPR groups * LVI, PNI, tumor differentiation and RAS status were not available for all patients; percentages reflect assessed cases. ^a^Mann-Whitney U Test,  ^b^Chi-square, ^c^T-test. AAPR: Albumin-to-alkaline phosphatase ratio; LVI: lymphovascular invasion; PNI: perineural invasion

Variables		All (n:182), n (%)	AAPR≤0.323 (n:87), n (%)	AAPR>0.323 (n:95), n (%)	Test statistic values	p-value
Age (median (min–max)) years.		57 (18–87)	57 (25–80)	57 (18–87)	3986.5	0.681^a^
Sex	Male	110 (60.4)	53 (61)	57 (60)	0.016	0.898^b^
	Female	72 (39.6)	34 (39)	38 (40)		
Performance score	PS-0	15 (8.2)	7 (8)	8 (8)	2.989	0.393^ b^
	PS-1	118 (64.8)	55 (63)	63 (66)		
	PS-2	34 (18.7)	20 (23)	14 (15)		
	PS-3	15 (8.2)	5 (6)	10 (11)		
Location of the tumor	Right	75 (41.2)	34 (39)	41 (43)	1.072	0.585^ b^
	Left	54 (29.7)	29 (33)	25 (26)		
	Rectum	53 (29.1)	24 (28)	29 (31)		
Lymphovascular invasion^*^	Yes	46 (25.3)	21 (55)	25 (45)	1.022	0.312^ b^
	No	48 (26.4)	17 (45)	31 (55)		
Perineural invasion*	Yes	39 (21.4)	16 (43)	23 (40)	0.120	0.729^ b^
	No	56 (30.8)	21 (57)	35 (60)		
Tumor differentiation*	Good	27 (14.8)	12 (29)	15 (25)	3.262	0.196^ b^
	Medium	64 (35.2)	23 (56)	41 (70)		
	Poor	9 (4.9)	6 (15)	3 (5)		
RAS status*	Wild	59 (32.4)	31 (74)	28 (52)	4.809	0.028^ b^
	Mutant	37 (20.3)	11 (26)	26 (48)		
Liver metastasis	Yes	112 (61.5)	63 (73)	49 (52)	8.991	0.003^ b^
	No	69 (37.9)	23 (27)	46 (48)		
Lung metastasis	Yes	41 (22.5)	21 (25)	20 (21)	0.340	0.560^ b^
	No	139 (76.4)	64 (75)	75 (79)		
Lymph node metastasis	Yes	63 (34.6)	31 (36)	32 (34)	0.76	0.783^ b^
	No	119 (65.4)	56 (64)	63 (66)		
Peritoneal metastasis	Yes	67 (36.8)	28 (33)	39 (41)	1.134	0.287^ b^
	No	112 (61.5)	56 (67)	56 (59)		
Number of metastases	≤2	34 (18.7)	14 (16)	20 (21)	0.736	0.391^ b^
	>2	148 (81.3)	73 (84)	75 (79)		
Presentation of the disease	De novo	162 (89)	83 (95)	79 (83)	6.961	0.008^ b^
	Recurrence	20 (11)	4 (5)	16 (17)		
Albumin (g/L), median (range)		36 (21-50)	35 (21-48)	36 (23-50)	-2.149	0.030^c^
ALP (U/L), median (range)		103 (38-1035)	165 (86-1035)	81 (38-123)	292.5	0.001^a^

The patients’ median ALB and ALP level was 36 (21-50) g/L and 103 (38-1035) U/L, respectively. The ROC analysis of the pre-treatment AAPR, NLR, and PLR for the PFS duration of first-line chemotherapy was insignificant (p = 0.335, p = 0.085, and p = 0.565, respectively). The pre-treatment ALB and ALP values ​​for PFS were insignificant in the ROC analysis (p=0.716 and p=0.188, respectively).

**Figure 1:**
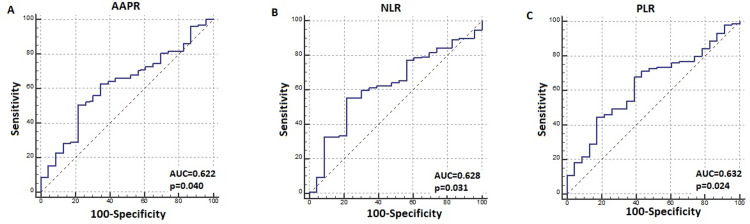
Receiver operating characteristics (ROC) analysis for overall survival; A) albumin-to-alkaline phosphatase ratio (AAPR), B) neutrophil-to-lymphocyte ratio (NLR), and C) platelet-to-lymphocyte ratio (PLR).

In the ROC analysis of the pre-treatment AAPR value for OS, the AUC was 0.622 (95% CI = 0.548-0.693) (p: 0.040), and the optimal cut-off AAPR value was ≤0.323 (Figure 1A). Accordingly, patients were categorized into low AAPR (≤0.323, n=87) and high AAPR (>0.323, n=95) groups. Significant differences were observed in RAS status, liver metastasis, and disease presentation characteristics between the two groups. The baseline characteristics of the two groups are compared in Table 1. The median AAPR was 0.30 (IQR: 0.18-0.43) and 0.36 (IQR: 0.26-0.49) in those with and without liver metastases, respectively (p=0.018). Although pre-treatment ALB levels did not differ significantly between these groups (p=0.339), pre-treatment ALP levels were significantly higher in patients with liver metastasis (p=0.012). Moreover, the AAPR was 0.32 (IQR, 0.20-0.43) in de novo metastatic patients and 0.48 (IQR, 0.36-0.53) in recurrent patients (p=0.001). ROC analysis of pre-treatment NLR and PLR showed significance for OS (AUC:0.628, p = 0.031) and (AUC= 0.632, p = 0.024), respectively, (Figures 1B-1C).

Survival analysis

**Figure 2:**
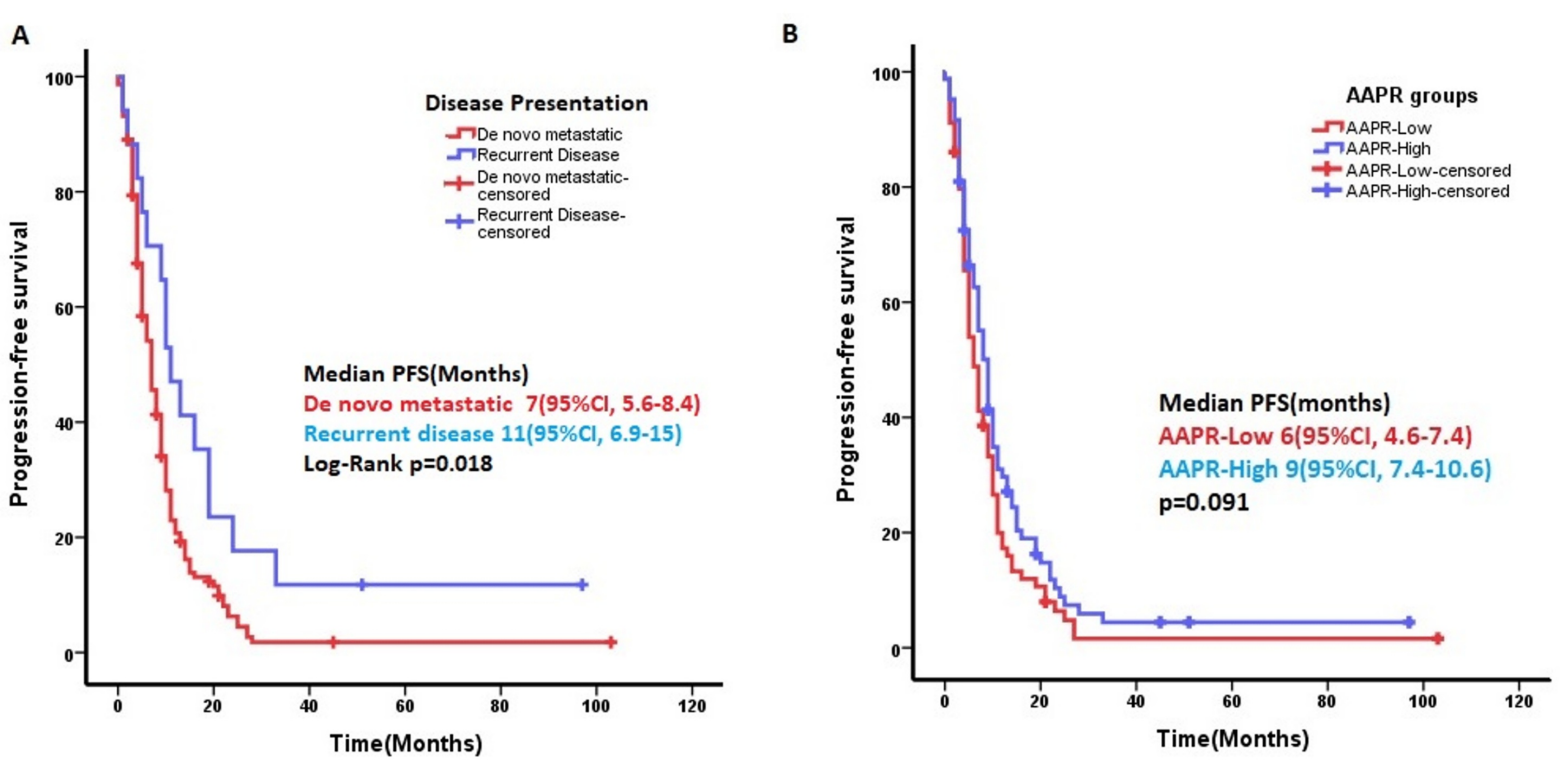
Progression-free survival (PFS) time was significantly longer in patients receiving chemotherapy due to recurrence compared to de novo metastatic patients (p=0.018). B) The difference between the PFS times of patients with low- and high-AAPR groups was not significant (p:0.091). AAPR: Albumin-to-alkaline phosphatase ratio

In 163 (90%) patients who received first-line systemic chemotherapy, the median PFS was seven months (95%CI: 5.6-8.3). Among these, 96 (53%) received FOLFOX plus anti-EGFR or anti-VEGF, and 31 (17%) received FOLFİRİ plus anti-EGFR or anti-VEGF treatment protocols. Univariate Cox regression analysis identified CA-19.9 level, neutrophil count, and presentation of the disease (de novo versus recurrence) as significant factors affecting PFS. In ROC analysis for PFS, the cut-off for CA-19.9 and neutrophil count was 9.35 U/mL (AUC: 0.732, p=0.006) and 5.67 103/µ (AUC: 0.674, p=0.038), respectively. However, Kaplan-Meier analysis revealed no significant difference in PFS between low and high CA-19.9 or neutrophil count groups (p = 0.187 and p = 0.486, respectively). Median PFS with first-line chemotherapy was seven (95%CI: 5.5-8.4) months in the de novo metastatic group and 11 (95%CI: 6.9-15) months in the recurrence group (p=0.018) (Figure 2A). There were no significant independent parameters for PFS in the multivariate analysis. In Kaplan-Meier analysis, median PFS was six (95%CI: 4.6-7.4) and nine (95%CI: 7.4-10.6) months in the low- and high-AAPR groups, respectively (p=0.091) (Figure 2B).

The median OS time for the entire cohort was 16 (95% CI: 12.7-19.3) months. The five-year survival rate of the patients was 12%. During the study period, 23 (12.6%) patients were alive, and 159 (87.4%) had died. Univariate Cox regression analysis identified LVI, tumor differentiation degree, PNI, peritoneal metastasis, AAPR, ALP, platelet, neutrophil, and monocyte counts ​​as predictors of OS. In univariable analysis, the AAPR for OS was significant (HR: 0.23, 95% CI: 0.08-0.62, p=0.004). Among the AAPR components, ALB was not significantly associated with OS in the univariate analysis (p=0.439), but ALP was significantly associated with OS in the univariate analysis (p<0.001). The univariate and multivariate analyses of the examined parameters are shown in Table 2.

**Table 2:** Parameters significantly affecting overall survival in univariable Cox regression analysis and a significant model was created with these parameters in multivariable Cox regression

		Univariable		Multivariable	
Variables		HR (95% CI)	p value	HR (95% CI)	p-value
Lymphovascular invasion (LVI)	No	Reference	0.023	Reference	0.307
	Yes	1.68 (1.07-2.63)		1.45 (0.71-2.95)	
Perineural invasion (PNI)	No	Reference	0.020	Reference	0.884
	Yes	1.71 (1.09-2.68)		1.06 (0.51-2.21)	
Tumor differentiation	Good	Reference	0.047	Reference	0.286
	Medium	1.22 (0.74-2.01)		1.24 (0.69-2.24)	
	Poor	2.98 (1.33-6.67)		5.44 (0.59-50.16)	
Peritoneal metastasis	No	Reference	0.004	Reference	0.047
	Yes	1.62 (1.17-2.23)		1.71 (1.01-2.92)	
Neutrophil count (10^3^/µ)		1.08 (1.04-1.13)	<0.001	1.01 (0.92-1.10)	0.825
Platelet count (10^3^/µL)		1.001 (1.000-1002)	0.012	0.999 (0.997-1.001)	0.474
Monocyte count (10^3^/µ)		1.89 (1.28-2.77)	0.003	2.14 (0.65-6.99)	0.210
Albumin-to-alkaline phosphatase ratio (AAPR)		0.23 (0.08-0.62)	0.004	0.555 (0.099-3.116)	0.504
Neutrophil-to-lymphocyte ratio (NLR)		1.004 (0.981-1.028)	0.718	-	-
Platelet-to-lymphocyte ratio (PLR)		1.000 (0.999-1.001)	0.403	-	-

**Figure 3:**
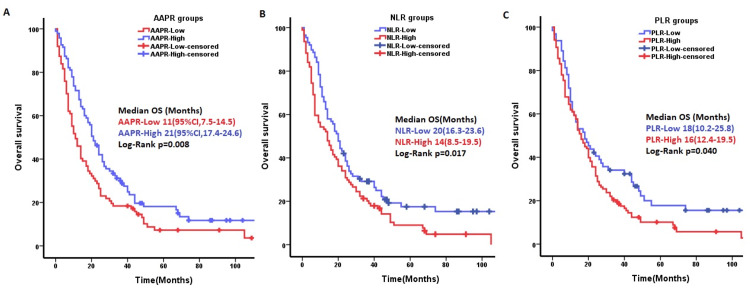
Kaplan-Meier curves of different rates for overall survival (OS). A) Overall survival of patients in the high-AAPR group was better than that of the low-AAPR group (p=0.008). B) Overall survival of patients in the low-NLR group was better than that of the high-NLR group (p=0.017). C) Overall survival of patients in the low-PLR group was better than that of patients in the high-PLR group (p=0.04). NLR: Neutrophil-to-lymphocyte ratio; PLR: platelet-to-lymphocyte ratio; AAPR: albumin-to-alkaline phosphatase ratio

The median OS of patients in the low- and high-AAPR groups was 11 (95%CI: 7.5-14.5) and 21 (95%CI: 17.4-24.6) months, respectively (p=0.008) (Figure 3A). The NLR and PLR were not significant for OS in univariable Cox regression. There was a difference in overall survival times between the low and high groups in the Kaplan-Meier analysis (p=0.017 and p=0.040, respectively) (Figures 3B-3C).

In the Kaplan-Meier analysis, parameters with OS differences in subgroups, such as degree of differentiation, LVI, PNI, peritoneal metastasis, presentation of the disease, AAPR, PLR, NLR platelet count, neutrophil count, and monocyte count, are summarized in Table 3.

**Table 3:** In ROC analysis, appropriate cut-off values were found for numerical variables. Patients below the cut-off value were included in the "low group", and patients above the cut-off value were included in the "high group". In the Kaplan-Meier analysis, the median OS times of the subgroups were summarized AAPR: Albumin-to-alkaline phosphatase ratio; NLR: neutrophil-to-lymphocyte ratio; PLR: platelet-to-lymphocyte ratio.

Variables	Cut-off value	Subgroups	Median OS (95%CI) months	p-value
AAPR	≤0,323	Low	11 (95%CI,7.5-14.5)	0.008
		High	21 (95%CI,17.4-24.6)	
Lymphovascular invasion (LVI)	-	No	31 (95%CI,15-46.8)	0.02
		Yes	21 (95%CI,16-26)	
Perineural invasion (PNI)	-	No	26 (10.1-41.9)	0.016
		Yes	22 (15.8-28.1)	
Peritoneal metastasis	-	No	19 (14.2-23.8)	0.003
		Yes	14 (9.0-18.9)	
Tumor differentiation	-	Good	24 (15.1-32.9)	0.017
		Medium	19 (12.0-25.9)	
		Poor	7 (4.1-9.9)	
Disease presentation pattern	-	De novo	14 (10.7-17.2)	0.016
		Recurrence	26 (17.2-34.7)	
Neutrophil count (10^3^/µ).	≤5.62 10^3^/u	Low	20 (15.6-24.4)	0.028
		High	13 (8.8-17.1)	
Monocyte count (10^3^/µ)	≤0.695 10^3^/u	Low	25 (20-29.9)	0.001
		High	12 (8.8-15.2)	
Platelet count (10^3^/µL)	≤286 10^3^/µL	Low	23 (17.2-28.7)	0.001
		High	13 (8.9-17)	
NLR	≤3.169	Low	20 (16.3-23.6)	0.017
		High	14 (8.5-19.5)	
PLR	≤148,8	Low	18 (10.2-25.8)	0.040
		High	16 (12.4-19.5)	

## Discussion

CRC is a common and important cause of cancer-related mortality. The prognosis of mCRC is affected by disease burden, disease biology, treatment options, and many patient-specific factors. This study investigated the prognostic value of an inexpensive and easily accessible AAPR in mCRC. This study found that the AAPR affected survival, similar to known factors, such as LVI, PNI, degree of differentiation, and peritoneal metastasis. The overall survival was significantly shorter in the low AAPR group than in the high-AAPR group (p=0.008). In the same patient group, the effect of the AAPR on survival was significant, whereas the effects of the NLR and PLR on survival were not.

ALB, a protein produced by the liver, is essential for maintaining plasma oncotic pressure. Hypoalbuminemia may be an indicator of liver synthesis dysfunction, kidney disease, malnutrition, and inflammation [11]. Fluorouracil, oxaliplatin, and irinotecan, used to treat CRC, often circulate in the plasma by binding to ALB. The efficacy of these drugs is affected in patients with hypoalbuminemia [12]. ALB is a negative phase reactant, and its synthesis is suppressed by inflammation and malignancy [13]. ALP is a hydrolytic enzyme complex involved in dephosphorylation and transphosphorylation. ALP is produced primarily in the liver, bones, and kidneys. ALP frequently increases cholestasis in the liver, bone, kidney, and biliary tract. High ALP levels are associated with cancer progression and poor survival [14]. ALP regulates inflammation and the immune system [15]. Decreased ALB and increased ALP levels are often associated with poor prognosis in patients with cancer. The AAPR index, obtained by dividing ALB by ALP, is a more objective indicator of malnutrition and cancer-related inflammation. NLR and PLR indices can be affected by all factors affecting the hematopoietic blood cell, such as infection and medication. Consequent to the effects on the hematopoietic blood cell, NLR and PLR indices can increase and decrease over time. The AAPR index, comprising albumin and alkaline phosphatase parameters, is less affected than the hematopoietic blood cell and increases and decreases over a longer period of time. Therefore, the AAPR index reflects survival in CRC better than the NLR and PLR indices [16]. In some cancers, a low AAPR level is associated with a poor prognosis. Cai et al. found a cut-off of 0.38 in 237 untreated patients with advanced hepatocellular carcinoma. In this study, the pre-treatment AAPR was prognostic, and the median OS was significantly shorter in the low AAPR group than in the high-AAPR group (2.4 vs 5.8 months, respectively) [9]. Li et al. found that the optimal cut-off for pre-treatment AAPR was 0.48 in 191 patients with metastatic gastric cancer. They found that pre-treatment AAPR was prognostic for OS (7.7 vs. 11.6 months) and PFS (4.3 vs. 8.6 months) in the low and high groups, respectively [8]. In a meta-analysis of 5716 patients with hepatocellular carcinoma, nasopharyngeal carcinoma, breast cancer, non-small cell lung cancer, and urothelial carcinoma, Xie et al. found that a low AAPR was associated with poor and short disease-free survival (p<0.001) [17]. In our study, the PFS of the pre-treatment AAPR-low group was shorter than that of the high-AAPR group, but the difference was not statistically significant (6 vs. 9 months, p=0.091). The PFS was longer in patients in the high-AAPR group, but statistical significance was not reached. Numerous factors influence PFS duration, including disease-related characteristics, patient characteristics, and treatment-related characteristics. Of these, PFS was shorter in de novo metastatic patients compared to metachronous metastatic patients, and there was a significant difference. The lack of significance of the AAPR index for PFS may be due to the numerous factors affecting PFS duration and the insufficient number of patients in our cohort to reveal these confounding factors. In other words, the predictive effect of the AAPR index for PFS was not significant, but its prognostic effect for OS was significant. Consistent with the literature, we found that the pre-treatment AAPR index was a factor that significantly affected OS in the univariate analysis of 182 patients with metastatic CRC, and OS was worse in the low AAPR group than in the high-AAPR group (11 vs.21 months, respectively, p=0.008). The AAPR for OS was significant in a univariable analysis but lost statistical significance in a multivariable analysis. This may be owing to the numerous factors that influence survival in mCRC, including disease burden, metastatic sites, disease grade, disease location, patient age, patient performance status, comorbidities, and treatments received. A larger patient population was needed to observe the effect of the AAPR among these factors. In our study, the number of patients with liver metastases was higher in the low-AAPR group than in the high-AAPR group. This was related to the fact that there was no difference in ALB levels between patients with and without liver metastases; however, ALP levels were significantly higher in patients with liver metastases. In patients with CRC and liver metastases, ALB production is affected, leading to lower ALB levels, whereas if bile ducts are affected, ALP levels are elevated. Consistent with this, our study found a significantly lower AAPR in patients with liver metastases.

The relationship between systemic inflammatory response indices and survival in patients with CRC and those with other cancers has been investigated. Neutrophilia is associated with tumor-related inflammation and angiogenesis and has been associated with poor survival in patients with CRC [18]. Thrombocytosis inhibits cytolysis of tumor cells by the immune system and has been associated with poor disease-free survival and OS in patients with CRC [19]. Pre-treatment lymphopenia reflects cancer-related immunosuppression and is associated with poor OS, poor PFS, and serious hematologic side effects of chemotherapy in patients with CRC [20]. Lymphocytes are important in antitumor immunity by inducing apoptosis and inhibiting tumor progression and have been associated with favorable survival in cancer [21]. The prognostic values ​​of neutrophilia, thrombocytosis, lymphopenia, and lymphocytosis are known; however, their ratios, such as the neutrophils-to-lymphocytes or platelets-to-lymphocytes ratios, are biomarkers that better predict survival in patients with cancer. He et al. compared NLR and PLR indices in 234 patients with mCRC. Patients with a high NLR or PLR had poorer outcomes regarding OS and PFS [22]. To the best of our knowledge, no studies to date have compared the prognostic significance of the AAPR with that of the NLR and PLR in patients with mCRC. In our previous study, we evaluated the prognostic value of the AAPR in a cohort of 540 patients with early-stage CRC using a similar study design and compared its performance with the NLR and PLR. The AAPR demonstrated significant prognostic value for OS, whereas the NLR and PLR did not. Recurrence and mortality rates were significantly higher in the AAPR-low group (p = 0.001). Furthermore, median disease-free survival (DFS) was significantly shorter in patients with a low AAPR (p = 0.015), and the AAPR was identified as an independent prognostic factor for OS (hazard ratio (HR): 0.42, p = 0.044). The optimal AAPR cut-off was 0.423 in patients with early-stage CRC and 0.323 in those with metastatic disease [23]. This difference may be related to the higher prevalence of hypoalbuminemia and elevated ALP levels in mCRC. Consistent with other studies, in our study, if the NLR and PLR were divided into low and high groups, OS was worse in patients in the high NLR or PLR groups in the Kaplan-Meier analysis. Our study found that the AAPR was significant for OS in the univariate analysis, whereas NLR and PLR were not significant in the same patient cohort. This may be because the AAPR better predicts OS, as changes in neutrophilia, platelets, and lymphocytes occur in the acute period during the cascade of inflammatory markers, whereas changes in ALB and ALP occur more frequently in the chronic period [24].

The cut-off values ​​found in studies conducted with hemogram-based parameters and those conducted with the AAPR vary among studies. The differences in these cut-off values ​​may be because the studies were conducted on different types of cancer, patients at different stages, and societies of different ethnic origins. In a meta-analysis by Lin et al., studies on patients with hepatocellular cancer, nasopharyngeal cancer, small cell lung cancer, renal cell cancer, non-small cell lung cancer, pancreatic cancer, and cervical cancer were included, and the AAPR cut-off was in the range of 0.23-0.68 [6]. The relatively low AAPR (0.23) in hepatocellular cancer can be explained by decreased ALB synthesis and increased ALP. In our study, the cut-off value for the AAPR in mCRC was 0.323.

Our study had some limitations. This was a retrospective, single-center study, which included a relatively small number of patients to fully control for confounding factors. There may be selection bias in retrospective studies. There is no standard cut-off for the AAPR value, and each study has determined a different cut-off value. The proportional hazards assumption for the Cox regression model was not formally tested, which should be considered a limitation of this study. Therefore, external validation is necessary to establish a standard cut-off value. However, to our knowledge, this is the first study to comprehensively analyze the prognostic impact of the AAPR index on OS in patients with mCRC. Additionally, our study compared the AAPR with previously investigated NLR and PLR systemic inflammatory indices in the same patient group.

## Conclusions

In conclusion, the AAPR is a simple, noninvasive, inexpensive, and accessible biomarker-based index. The prognosis of mCRC is affected by disease burden, disease biology, treatment options, and many patient-specific factors. The AAPR index was not significantly associated with sex, age, tumor location (right- vs. left-sided colon; colon vs. rectum), or histopathological features, including lymphovascular and perineural invasion. The AAPR index was lower in patients with liver metastases than in those without a and in those with de novo metastatic disease than in those with recurrence. In patients with mCRC, OS was shorter in those with low AAPR than in those with a high AAPR. The AAPR showed a stronger univariable association with OS than the NLR/PLR in this cohort but did not retain independent significance in multivariable analysis; prospective validation is needed. 
